# Status of Novel Coronavirus Disease 2019 (COVID-19) and Animal Production

**DOI:** 10.3389/fvets.2020.586919

**Published:** 2020-11-05

**Authors:** Patrick Brice Defo Deeh, Veysi Kayri, Cemal Orhan, Kazim Sahin

**Affiliations:** ^1^Department of Animal Biology, Faculty of Science, University of Dschang, Dschang, Cameroon; ^2^Department of Animal Production and Technologies, Faculty of Applied Sciences, Muş Alparslan University, Muş, Turkey; ^3^Department of Animal Nutrition, Faculty of Veterinary Medicine, Firat University, Elazig, Turkey

**Keywords:** COVID-19, infection, pandemic, animal production, economy

## Abstract

In December 2019, a severe acute respiratory syndrome coronavirus-2 (SARS-CoV-2) that caused severe disease clusters was first reported in Wuhan, the capital of China's Hubei province. This viral disease, which is reported to originate from a seafood market where wild animals are illegally sold, has been transmitted among humans worldwide through close contact. Given the growing number of infected people worldwide and the disastrous consequences in all aspects of life, COVID-19 is a serious public health issue that requires special attention. In some countries, the epidemic curve of infection which was in the plateau phase or decreasing phase during the lockdown period increases day by day since the reopening, indicating the second phase of contamination. Therefore, the preventive measures recommended by the World Health Organization (WHO) must be respected to stop the spread of the disease. The international crisis due to the COVID-19 pandemic negatively affects many sectors, including animal production and its related industries. Indeed, with the cessation of imports and exports between countries, it is not possible to provide feeds that are considered as basic raw materials in livestock raising. This situation impairs animal movements, decreases production inputs availability, and negatively affects the economy. The sustainability of animal production is also affected by a shortage of workers due to the lockdown/curfew, the strong decrease in the purchasing power of the consumer, and the intensification of health care tasks. To prevent contamination of animal products and the spread of the disease with food, the U.S. Centers for Disease Control and Prevention (CDC) recommends frequent disinfection of food and human contact surfaces at production sites using an appropriate antiseptic. The purpose of this review article is to describe the current status of COVID-19 and investigate its effects on animal production. We propose potential approaches to keep animal products processing units and staff safe from SARS-CoV-2 infection and some strategies to improve animal production quantity and economy.

## Introduction

Virology is the science that studies viruses that can be transmitted by food, soil, air, and contact (1, 2). Viral infections whose cause of occurrence and their effects on human health are not fully known to have threatened mankind throughout history. The COVID 19 pandemic, which affects the whole world and infects 32.23 million people as of September 25, 2020, is a serious public health issue. Coronaviruses from the Corononaviridae family are enveloped positive polarity and single-chain RNA viruses that can infect both humans and animals (3, 4). However, coronaviruses are divided into four groups as alpha, beta, gamma, and delta coronaviruses. Coronaviruses that can be transmitted to humans are alpha and beta coronaviruses (5). To date, six coronaviruses (CoVs HCoVs-NL63, HCoVs-229E, HCoVs-OC43, HCoVs-HKU1, SARS-CoV, and MERS-CoV) which can infect humans have been identified (4, 6).

In December 2019, an unknown epidemic of lung infections occurred in Wuhan, China, and spread to the whole country in a short period of 2–3 weeks. It was determined that the pathogen causing this epidemic was a coronavirus and the International Virus Taxonomy Committee named the severe acute respiratory syndrome coronavirus-2 (SARS-CoV-2), based on taxonomy (7, 8). This new type of beta coronavirus emerged in a market selling seafood in Wuhan and it was determined that 65% of the 41 confirmed cases were directly or indirectly associated with this market place (9). It is found that the disease is transmitted from person to person when it is also seen in people who have no connection with the market place, where the seafood is sold (10). The incubation period of COVID-19 is on average 5 to 6 days, however, can be up to 14 days in some cases (11). Clinical signs such as cough, fever, runny nose, and fatigue are observed in infected patients (12). However, asymptomatic patients have no symptoms of the disease. According to the studies, based on the previous coronavirus outbreaks and the genetic structure of the existing virus, it is thought that COVID-19 may have been transmitted from bats (13), or pangolins since the virus taken from pangolin are 99% of the genetic similarity of the virus taken from humans (14). The main way of transmission of COVID-19 is thought to be that the droplets that come out of the mouth of infected people during the speech, coughing, sneezing settle in the upper respiratory tract of other people, and then reach the lungs and cause infection. Another way of transmission is thought that people who touch the coronavirus-infected surfaces with their hands touch their hands to their mouths, noses, and eyes and take the disease factor (10, 15).

The COVID-19 pandemic has direct impacts on food systems, especially through changing the food supply-demand system, and indirect impacts through decreasing purchasing power and the capacity of food distribution and marketing, and increasing healthcare tasks (16). For instance, the disruptions of production and industrial supply chains due to the COVID-19 crisis are expected to reduce the global economic growth by 0.5% in 2020 (2.4% in 2020 vs. 2.9% in 2019) (17). These elements may increase the risk of food insecurity, malnutrition, and poverty in the countries most affected by the crisis (18). Given the growing number of infected people worldwide and the disastrous consequences in all aspects of life, COVID-19 is a serious public health issue, which requires special attention. The purpose of this review article is to describe the status (epidemiology, symptoms, diagnosis, and treatments) of COVID-19 and elucidate approaches to prevent contamination during animal production and boost the economy.

## General Information on SARS-CoV-2

### Epidemic Curve of Infection

The epidemic curve of infection is generally divided into 3 parts: increasing, plateau, and declining phases (16). The 3 phases of the epidemic curve of all countries are different. In many countries such as USA, Brazil, and India, the peak of infection is not yet observed. Some countries like the United Kingdom, Canada, France, and Italy, which were in the decline phase of the disease on Jun 21, 2020 (19), have cumulative confirmed cases, which continue to increase as of July 21, 2020 (19). This difference could be due to many factors such as population size, the average age, the implementation of some preventive measures, the equipment of hospitals, and the percentage of peoples with chronic diseases which may increase contamination and mortality. It is important to note that the second phase of contamination is observed in some countries like Brazil, the United States, Turkey, France, Australia, UK, and Spain probably due to the reopening (19).

**Figure 1:**
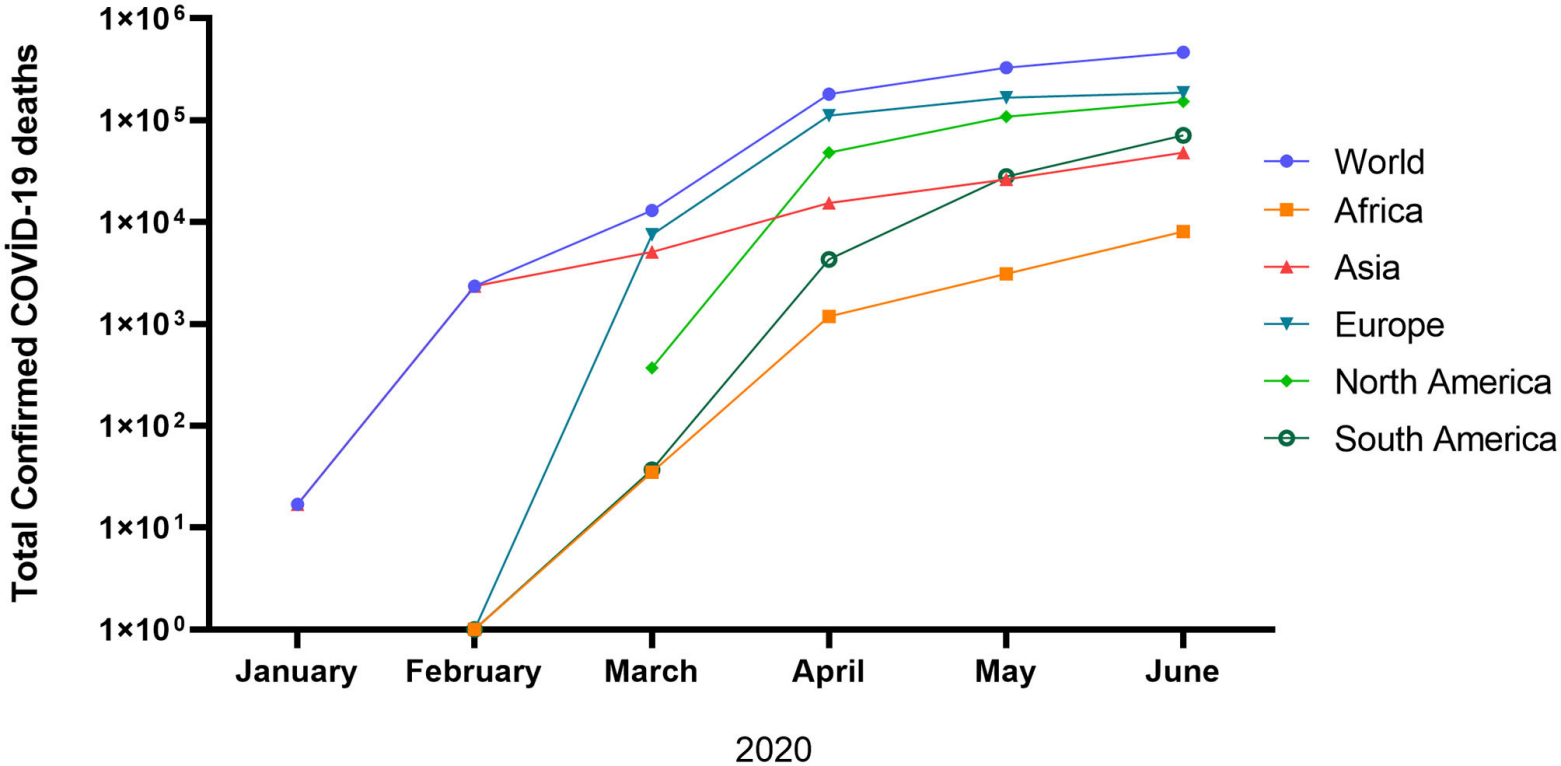
Total confirmed COVID-19 deaths (17).

In general, the peak of infection and deaths in the world is not yet observed. The total confirmed COVID-19 cases and deaths are 14.89 million and 610097, respectively as of July 22, 2020 [(19); Figure 1]. The high mortality of infected patients in Europe and the North, America could be due to the high average age and associated chronic diseases, which may decrease the immunity system (20–22). Although the cumulative confirmed COVID-19 deaths are still increasing in the United Kingdom, France, United States, Brazil, Canada, and India, a plateau phase is observed in Germany (19). The lowest death rate in Germany could be justified by the effective response to COVID-19 by implementing mass testing and swift lockdown associated with the robust healthcare system of the country. To date (as of September 25, 2020), the current status of COVID-19 in the world is still worrying (32.23 million positive cases, 983 042 deaths, and 22.24 million recovered).

### Symptoms and Diagnosis

People infected with COVID-19 can be asymptomatic or present various symptoms such as fever, cough, dyspnea, fatigue, muscle or body aches, headache, loss of taste or smell, congestion or runny nose, nausea, vomiting, and diarrhea (23, 24). COVID-19 can affect the neurological system and cause multiple organ failure such as kidney failure. In addition, pre-existing comorbidities such as heart disease, diabetes, hypertension, respiratory disorders, and cancers increase the severity of COVID-19 in patients (25, 26).

At the beginning of this pandemic, due to the lack of specific SARS-CoV-2 kits, the WHO has released a guideline on case surveillance of COVID-19 on January 31, 2020 (27), which recommended to first diagnose the most common respiratory diseases, followed by SARS-CoV-2 detection, if the previous exams are negative (28). Although quarantine of suspect patients with some symptoms of the disease was also recommended in many countries at the beginning of this pandemic, systematic diagnosis of SARS-CoV-2 should be a priority to control the spread of this pathogen. For SARS-CoV-2 testing, the World Health Organization (WHO) strongly recommends the collection of samples from respiratory tract specimens for the detection of viral nucleic acid (29). The real-time RT-PCR assay is the first line of molecular-based methods used, due to its high sensitivity (30–32). However, if a negative result is found in patients suspected of COVID-19 (presented classical symptoms of the disease), it is advisable to repeat the test (33). Although the effectiveness of the real-time RT-PCR assay is proven, it requires sophisticated equipment and highly trained personnel and is relatively time-consuming (about 1.5–2 h) (34). Recently, new rapid techniques such as rapid antigen testing serological tests, reverse transcription loop-mediated isothermal amplification (RT-LAMP), and CRISPR-Cas12-based assay called SARS-CoV-2 DNA endonuclease-targeted CRISPR Trans Reporter (DETECTR) have been developed for rapid and sensitive detection of SARS-CoV-2 (35).

### Therapy

#### Modern Treatment

There is no specific therapy for COVID-19 and no effective vaccine is available (36). However, depending on the stage of the disease and comorbidity factors, the treatment of COVID-19 patients included antimalarial drugs, antibiotic therapy, antiviral treatment, corticosteroids, immunoglobulin therapy, and convalescent plasma from patients recovered from COVID-19 (37–40).

A combination of hydroxychloroquine (an antimalarial drug) and azithromycin (an antibiotic agent) has been approved to be effective in many countries, based on the works conducted in China (37, 41) and France (42). The effectiveness of chloroquine on COVID-19 infection has been reported *in vitro* (32). A clinical study conducted in more than 10 hospitals in Wuhan (China) also demonstrated the efficacy and safety of chloroquine phosphate in the treatment of COVID-19 associated with pneumonia (41). This antiviral property of chloroquine was already reported 17 years ago (43). Indeed, Savarino et al. (44) demonstrated that chloroquine has antiviral potential by increasing endosomal pH required for virus/cell fusion, and interfering with the glycosylation of cellular receptors of SARS-CoV. The *in vitro* antiviral activity of chloroquine has been also reported on other viruses such as Middle East Respiratory Syndrome Coronavirus (MERS-CoV), HIV, Ebola, Hendra, and Nipah (43–46).

Antiviral drugs such as favipiravir (an anti-influenza agent), remdesivir (an anti-ebola drug), lopinavir and ritonavi (commonly used against HIV) and pegylated interferon with ribavirin (used in the treatment of HBV and HCV) were urgently administered to patients with COVID-19 (32, 33, 47, 48). In a recent study (without a control group) conducted on 53 COVID-19 patients under remdesivir therapy, a clinical improvement was observed in 36 (68%) patients (49), suggesting the beneficial potential of this protease inhibition in the treatment of COVID-19. Oseltamivir also improved the clinical symptoms of patients with severe COVID19 in 35.8% (393/1099) (39), 89.9% (124/138) (9), and 92.7% (38/41) (38) patients. Although an *in vitro* study demonstrated the inhibitory potential of lopinavir and ritonavir against SARSCoV (50), no clinical improvement was observed in 199 patients with severe COVID-19 treated with these drugs (51). Clinical studies to identify effective antiviral drugs for the treatment of COVID-19 are underway.

The use of serum or convalescent plasma from patients recovered from COVID-19 represents an effective treatment strategy because the treated patients from the viral infection produced a specific antibody in response to SARS-CoV-2 (52). Although the use of corticosteroids has been reported as an effective treatment of COVID-19 (41), their use is controversial. 30 clinical trials have been initiated to date (as of September 24, 2020) to evaluate the safety and efficacy of dexamethasone against SARS-CoV-2 infection^1^. Dexamethasone has been proven to significantly reduce the mortality risk in COVID-19 patients on ventilation and oxygen by up to 35 and 20%, respectively. Based on positive results, dexamethasone has been authorized by the UK government for the treatment of critically ill COVID-19 patients (53). However, the comorbidity factors (especially diabetes) should be considered before treatment because some researchers reported that dexamethasone increases the risk of COVID-19 progression to severe disease, especially in patients with diabetes (54, 55).

#### Traditional Treatment

In China, in a total of 701 patients with severe COVID-19 treated with traditional Chinese medicine based on *Qingfei paidu* decoction, a clinical improvement was observed in 449 patients while 212 cases were stable (56). An important study carried out on Vero E6 cells by Runfeng et al. (57) demonstrated that Lianhuaqingwen (a traditional Chinese medicine formula) may represent a novel strategy to control COVID-19 because it significantly inhibits the SARS-COV-2 replication, affects virus morphology and exerts anti-inflammatory activity *in vitro*. Other Chinese plants like *Astragulus membranaceus* and *Pelargonium sidoides* commonly used to treat viral respiratory infections (58, 59) may also be useful against COVID-19.

The anti-mouse coronaviral activity (a surrogate of SARS-CoV) of some Indian plants including *Indigofera tinctoria, Vitex trifolia, Gymnema sylvestre, Abutilon indicum, Leucas aspera, Cassia alata, Sphaeranthus indicus, Clerodendruminerme gaertn*, and *Evolvulus alsinoides* have been reported (60). These Indian plants may represent a potential therapy of COVID-19. More experimental and clinical studies are highly needed.

In Cameroon, many plants such as *Aloe vera, Angelica gigas, Panax ginseng, Scutellaria baicalensis, Trichilia dregeana, Detarium microcarpum, Phragmanthera capitate*, and *Phyllanthus muellerianus* have been reported to exhibit antiviral and immunomodulatory properties through stimulation of cytokines, activation of lymphocytes and improvement of macrophage actions (61, 62). These plants may represent a possible source of COVID-19 therapy.

In Madagascar, a polyherbal formulation called COVID Organics (CVO) composed of *Artemisia afra* and other Madagascan plants has been recommended by the President of the Republic for the prevention and treatment of COVID-19. The consumption of CVO by the Madagascan population may justify the low prevalence of COVID-19 in the country (8,162 positive cases, 69 deaths, and 4,662 recovered as of 22 July 2020). However, experimental and clinical studies are underway by international researchers to confirm the effectiveness of CVO for the treatment of COVID-19. Researchers at Germany's Max Planck Institute of Colloids and Interfaces in Potsdam are among a group of scientists from Germany and Denmark in collaboration with the US company ArtemiLife to conduct a project on the effects of *Artemisia annua* on COVID-19 (63). On the other hand, Eucalyptus essential oil found in many countries is reported to improve the innate cell-mediated immune response that can be used as an immunoregulatory agent against infectious diseases (63, 64).

#### Nutritional and Dietary Therapy

The immunomodulatory potential of some nutrients has been proposed as a possible strategy to prevent and/or treat SARS-CoV-2. For instance, since vitamin C (ascorbic acid) has been used for decades in the treatment of influenza (a coronavirus) (65), it may also be effective against SARS-CoV-2, but clinical studies are needed. Vitamin D intake may reduce the risk of COVID-19 infection and related deaths. Indeed, evidence has shown that vitamin D decreases the risk of COVID-19 outbreak in winter, which is a time when 25-hydroxyvitamin D (25(OH)D) level is low (66). Vegetables rich in vitamin A like carrots, spinach, and sweet potato may also be helpful against COVID-19 infection. Vitamin A comprises a group of fat-soluble compounds (including retinol, retinoic acid, and β-carotene) known to lower the susceptibility to infections (67). For example, isotretinoin (a derivative of vitamin A) mediates the down-regulation of angiotensin-converting enzyme 2 (ACE2), which is a crucial host cellular protein required for the entry of SARS-COV-2 in the body (68). Rice bran, wheat bran, *Lawsonia alba* (hina), *Echinacea purpurea* (eastern purple coneflower), *Plumbago zeylanica* (Ceylon leadwort), and *Cissampelos pareira Linn* (velvetleaf) also exhibit immunomodulatory properties by stimulating phagocytosis (63, 64). Using these immunomodulatory vitamins and foods could improve the immune system and protect the body against COVID-19. However, experimental and clinical studies are needed to confirm their effectiveness.

## SARS-CoV-2 and Food

### Availability and Contaminated Ways of SARS-CoV-2 in Food

Although there is sufficient information about the direct transmission of the new type of beta coronavirus agent, the information about the indirect transmission is not clear. Although there is no evidence that the disease agent is contaminated with food in the studies conducted up to this time, it is known that there is a possibility of contamination from the virus-contaminated surfaces. Based on this information, an idea can be made about how the disease can be transmitted from food contaminated by the disease. In the statement published by the FDA (US Food and Drug Administration) in February 2020, there was no evidence that COVID-19 could be contaminated with consumed food and nutrient packaging, but it has been stated that it is always important for general hygiene and sanitation to be thoroughly disinfected the hands and surfaces when handling food, cooking, and serving. Raw meat products should not be kept together with other foods, and food should be cooked at the right temperature^2^.

According to the report published by the WHO, there is no information that the SARS-CoV-2 virus has been transmitted by foods, but according to the previous coronavirus outbreaks (SARS-CoV and MERS-CoV), it has been reported that there are some suspicions about the coronavirus can be found in animal foods that have not been heated treatment (20). This shows that animal products at processing plants, slaughterhouses, and butchers can play an important role in spreading the disease agent. According to another study, the emergence and spread of the disease factor in a sea market, where animal products are sold reveal that hygienic measures are very important (69). Moreover, a study reported that viruses that can be transmitted through the respiratory tract remain viable for up to 48 h in fruits and vegetables stored in the cold (70). Technological processes such as heating and cooling applied to prevent microbial growth in foods can be applied to block the reproduction of bacteria. However, if the contaminated agent is a virus, the aim is not to prevent development, but to destroy the virus completely (71).

It has been reported that coronaviruses remain stable in cold environments (71). It is also known that coronaviruses maintain their viability on different surfaces for 3–4 days at appropriate humidity and temperature parameters, and are sensitive to heat treatment at 70°C. Therefore, it has been reported by the WHO that meat, milk, and other animal products should not be consumed raw and contact with other foods should be avoided to prevent cross-contamination (72). Moreover, China's Disease Control and Prevention Center have determined that there is COVID-19 nucleic acid in 33 of 585 environmental samples collected in Wuhan province, China, where the disease originated (73).

### Making Animal Products Processing Units and Staff Safe From SARS-CoV-2 Infection

The COVID-19 outbreak has raised concerns about potential medical supply issues, including both pharmaceuticals and medical products such as personal protective equipment (e.g., gloves, masks, gowns) and surgical drapes (74). When working in the food industries, basic hygiene measures should always be implemented. The U.S. Centers for Disease Control and Prevention (CDC) recommends frequent disinfection of food and human contact surfaces at production sites. It states that the disinfection of these surfaces should be done like routine cleaning, no additional disinfectants are required. During the new type of beta coronavirus pandemic, it recommends that employees working in food businesses stay home if they show symptoms of the disease and not go out and go to work before the disease symptoms disappear completely (75). To prevent contamination of animal products with disease agents and the spread of the disease with food, the disinfection of the hands should be ensured during animal product processing, and the products should be consumed after being washed thoroughly or disinfected with an appropriate antiseptic. Animal products should not be consumed raw and they should be cooked at least at 70°C for a sufficient period. The devices should be cleaned and disinfected frequently. It is also recommended that the employees working in the animal products processing facilities should be clean during the production, stay at least 2 meters away from each other and disinfect their hands and clothing after leaving the production facility (76). Hence, biosecurity and biosafety should be improved in order to prevent the spread of this pathogen.

### Detection of SARS-CoV-2 in Companion and Wild Animals

A small number of animals worldwide have been reported to be infected with SARS-CoV-2, mostly after close contact with people infected with COVID-19. In March 2020, two dogs (in Hong Kong) and two domestic cats (in Italy and Hong Kong) belonging to SARS-CoV-2 infected persons and exhibiting COVD-19 symptoms were tested positive for SARS-CoV-2 (77–80).

On March 27, 2020, a four-year-old tiger at the Bronx Zoo in New York City developed a dry cough and some wheezing. On April 2, 2020, the samples were collected from the tiger and she was tested positive for SARS-CoV-2 (81). By April 3, three additional tigers and 3 lions in the same Zoo exhibited cough and a loss of appetite (important symptoms of SARS-CoV-2), but were not tested. These animals were immediately isolated. As of April 6, 2020, the presumably infected animals were stable and recovered, although they had not been tested (81).

Presently (as of September 24, 2020), the United States Department of Agriculture (USDA) has reported numerous cases of SARS-CoV-2 in cats, dogs, lions, tigers, and minks^3^.

### Veterinary Diagnosis, Surveillance, and Monitoring of Animals for SARS-CoV-2

Although routine testing of animals for SARS-CoV-2 is not recommended, adequate measures (testing, isolation, and treatments) must be implemented immediately in the case of suspected cases (animals or companion animals), using a One Health approach between local, state, and/or federal public health and animal health officials, as recommended by the Centers for Disease Control and Prevention, US Department of Agriculture^4^.

Adequate surveillance is needed to detect infected animals and workers at an early stage for efficient response to prevent the spread of disease. The WHO in cooperation with the Food and Agriculture Organization of the United Nations and OIE-World Organization for Animal Health encouraged collaboration, networking, and technical consultation for jointly analyzing epidemiological and human-animal interfaces and promptly sharing and distributing public health information (82).

### The Effects of SARS-CoV-2 on Animal Production Quantity and Economy

#### Effects on the Prices of Animal Feeds

The severity of the effects of the COVID-19 pandemic on agriculture, food, and therefore feed sector varies by country (83). Many factors, such as the measures taken by countries against the COVID-19 pandemic, the power of the measures taken, the economic structure of the countries, and the level of awareness of the society in terms of the epidemic, have been effective. Failure to import and export the feeds, which are the raw materials of livestock raising, and the continuity of animal production harm the narrowing of the market of animal products and the cost of the products. The mixed feed industry is an intermediate industry branch, but it takes its raw materials mostly from crop production and gives its final product to animal production. The effects of the COVID-19 pandemic on the mixed feed industry are seen as the problems experienced in the supply of raw materials and the supply of mixed feed demand (83).

**Figure 2:**
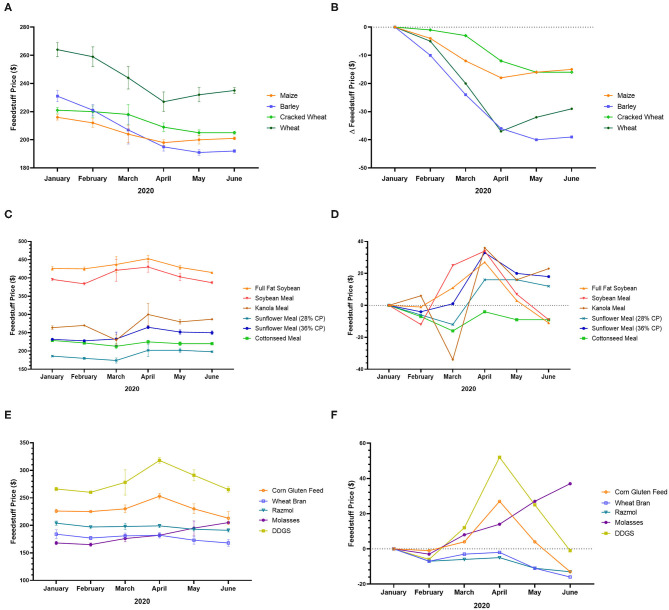
Feedstuff price variation in Turkey during the COVID-19 pandemic. **(A)** Price of maize, barley, cracked wheat, and wheat from January to June 2020. **(B)** Variation of the price of maize, barley, cracked wheat, and wheat from January to June 2020. **(C)** Price of full-fat soybean, soybean meal, canola meal, sunflower meal, and cottonseed meal from January to June 2020. **(D)** Variation of the price of full-fat soybean, soybean meal, canola meal, sunflower meal, and cottonseed meal from January to June 2020. **(E)** Price of corn gluten feed, wheat bran, rasmol, molasses, and DDGS from January to June 2020. **(F)** Variation of the price of corn gluten feed, wheat bran, rasmol, molasses, and DDGS from January to June 2020 (83).

For instance, in Turkey, the price of maize, barley, cracked wheat, and wheat decreased from January to June 2020 (Figures 2A,B). During the same period, the price of full-fat soybean, soybean meal, canola meal, sunflower meal, cottonseed meal, corn gluten feed, wheat bran, rasmol, molasses, and dried distillers grain with soluble (DDGS) was also unstable (Figures 2C–F). For instance, the price of DDGS, which was almost stable at the beginning of the pandemic (from January to February 2020) increased from February to April, after which it began to decrease and reach the baseline value in June 2020 (Figures 2E,F), indicating a balance between production and consumption. In addition, the price of molasses has continued to increase to date, while the prices of wheat bran and rasmol continue to decrease (Figures 2E,F), indicating high demand for molasses and low production, as well as low demand for wheat bran and rasmol, which reduced sales and lowered prices^5^.

**Figure 3:**
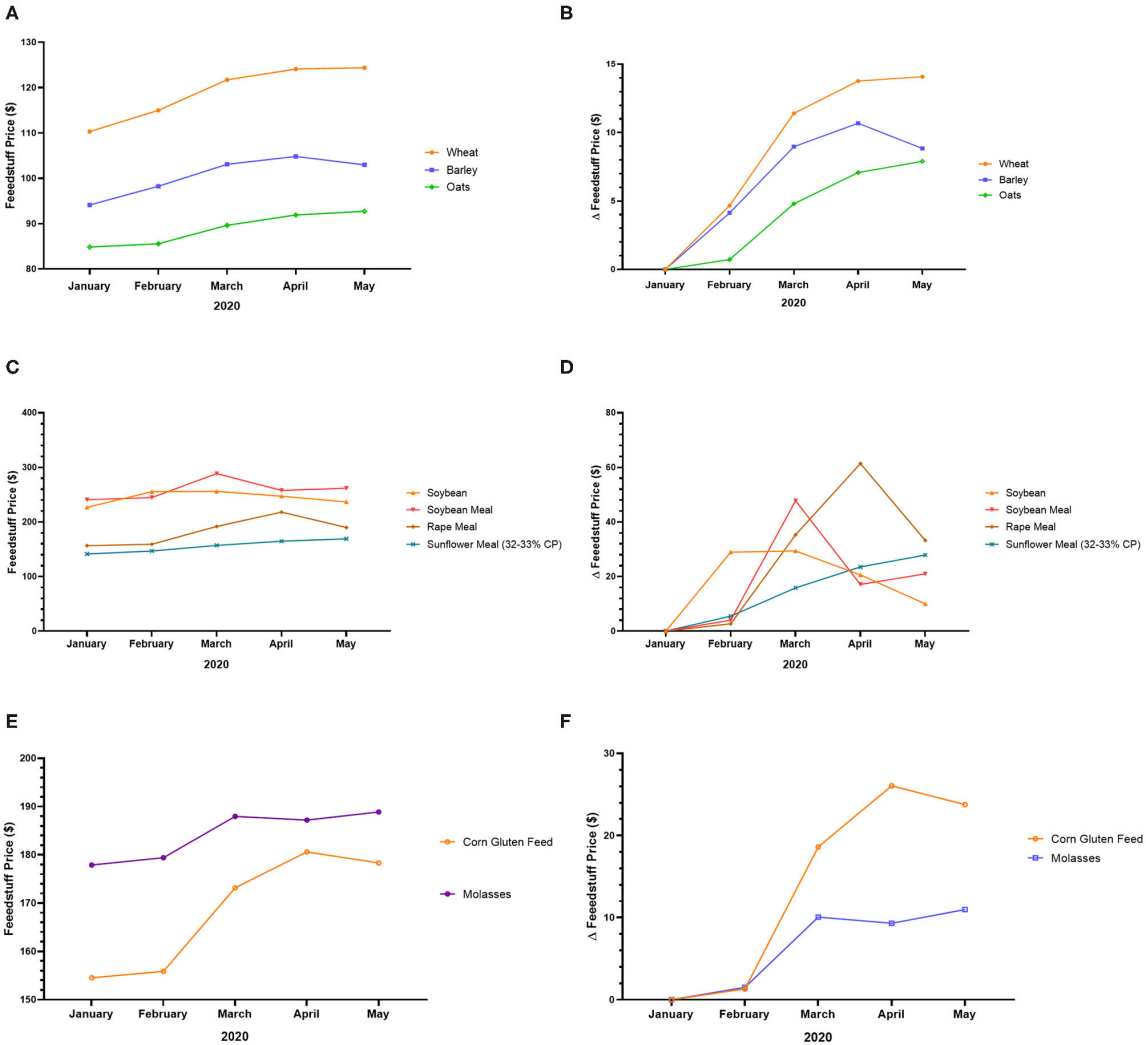
Feedstuff price variation in the UK during the COVID-19 pandemic. **(A)** Price of wheat, barley, and oats from January to May 2020. **(B)** Variation of the price of wheat, barley, and oats from January to May 2020. **(C)** Price of soybean, soybean meal, rape meal, and sunflower meal from January to May 2020. **(D)** Variation of the price of soybean, soybean meal, rape meal, and sunflower meal from January to May 2020. **(E)** Price of corn gluten feed and molasses from January to May 2020. **(F)** Variation of the price of corn gluten feed and molasses from January to May 2020^5^.

In the UK, the COVID-19 crisis increased the price of feedstuff. Indeed, the price of wheat, barley, oats, soybean, soybean meal, rape meal, sunflower meal, corn gluten feed, and molasses increased from January to May 2020, with a peak generally observed in March-April (except wheat, oats, and sunflower meal) (Figures 3A–F). The peak prices of certain feedstuffs such as wheat, oats, and sunflower meal have not yet been observed (Figures 3A–D). The rising price of feedstuff in the UK may be due to low production and high demand^6^.

Rations cannot be prepared as desired due to the inability to import feed. The basic principle taken into account when preparing the ration is to give animals the carbohydrates, protein, fat, vitamins, and minerals they need to obtain maximum efficiency with minimum cost. Productivity per animal is likely to decrease due to the lack of basic nutrients in animal feed. According to a study with 1,008 participants in Wuhan Province of China during the beginning of the disease, it has been reported that people's interest in game meat has decreased and the demand for organically produced animal products in farms has increased (84).

#### Effects on the Transport of Animal Feeds and Animal Production Products

During the COVID-19 pandemic, the transportation of animal feeds and food, including animal production products, encountered many obstacles. For instance, in China, milk transport was disrupted by tight road traffic controls, leading to milk dumping (85). Moreover, import and export difficulties by maritime transport, linked to several factors such as lack of manpower in Chinese ports, minimum time at sea imposed by certain ports on ships from China seriously disturbed the transport of animal products between China and neighboring countries. In the Philippines, the risk of a shortage of meat caused by the delay in vehicles transporting raw materials for processing has been avoided by stopping movement restriction. Within the countries of the European Union, 35% of beef is exported between member countries. Export bans have caused prices to plummet in Poland, with domestic consumption representing only 15% of production. In Latin America, especially in Argentina and Uruguay, restrictions on the transportation of animal products and the export of meat have lowered the incomes of farmers and ranchers, plunging many into unemployment. In West Africa, the restriction of animal feeds import due to the COVID-19 crisis decreased poultry and pig productions (86, 87).

#### Effects on Animal Disease Prevention and Control

The impact of the COVID-19 pandemic on animal disease prevention and control is observed around the world. Some examples are summarized in Table 1.

**Table 1:** The effects of COVID-19 on animal disease prevention and control.

**Area**	**Effects**	**Examples**
Farm	- Animal health activities such as diagnosis, vaccination, and medical treatments are not carried out adequately. - Overstocking of animals at the farm could increase stress and animal diseases, leading to high mortality, poor production performance, and economic losses. - The farms are not visited regularly by veterinary professionals. - In the case of animal disease, farmers are less likely to reach out to their veterinarians.	- Some animal drug companies that produced active pharmaceutical agents in China for the market of the United States of America have indicated that the supply chain disruptions caused by the COVID-19 crisis could soon lead to shortages (88).
Laboratory testing	- Reduced testing and diagnostic capacities due to the reduction in the number and availability of veterinarians, societal lockdowns as well as the lack of financial means and laboratory agents. - The difficulties in transporting samples from farms to laboratories and the lack of personal protective equipment (PPE).	- The high demand for COVID-19 testing has caused a shortage of agents and PCR equipment for collecting samples and extracting RNA (89–91). - According to WHO (92), the transmission of animal diseases has been increased in many countries due to the non-compliance of safety instructions and the lack of PPE.
National animal health activity	- The economic crisis caused by the COVID-19 pandemic may affect animal disease control programs. - Diagnostics, vaccination campaigns, and treatment of animal diseases cannot be implemented as planned due to movement restrictions.	- In Australia, the minister of Agriculture reported that movement restrictions due to the COVID-19 pandemic would delay the government response to an outbreak of African swine fever, regarding the early detection of the virus (93). - In Papua New Guinea, it is difficult to control African swine fever due to movement restriction caused by the COVID-19 crisis.
International animal health activity	- The activities of international organizations may be delayed or suspended due to the lack of funding and logistical support. - International animal health activities cannot be implemented as planned.	- FAO conference on “African swine fever unprecedented global threat: a challenge to food security, wildlife management, and conservation” has been postponed due to the COVID-19 lockdown (87). - FAO projects are delayed or postponed due to COVID-19 lockdown and the lack of financial supports.

#### Effects on the Economy of Animal Products and Strategic Future After the COVID-19 Outbreak

The closure of markets during the COVID-19 crisis created an imbalance between production and consumption, leading to lower demand, lower sales, and falling prices. For instance, American pig prices dropped by about 27 percent in just over a week^7^. The movement restriction implemented in many countries, the reduction in the number of workers employed in animal production facilities (due to the quarantine of infected or suspected workers) negatively affect the production capacity and contribute to the economic failure. Elleby et al. (83) perform a scenario-based analysis on the IMF economic growth forecasts for 2020 and 2021 using a global multi-commodity agricultural market model and concluded that a decline in economic growth will decrease international meat prices by 7–18% in 2020 and dairy products by 4–7% compared to a business as usual situation.

As reported by Hafez et al. (94), the strategic future after the COVID-19 outbreak should be mainly focused on disease control. For instance, in the poultry industry, eradication, elimination, and/or control of foodborne and zoonotic pathogens present a major challenge. Also, the countries should synchronize their legislation linked to the market, animal nutrition, and the drugs and vaccines licensing for veterinary practice, particularly after the COVID-19 pandemic (94).

#### Recommended Actions

The impact of the COVID-19 crisis on animal production is serious. The maintenance of animal production activities should be seen among the priority sectors and the decisions of the governments should be in this direction. Some recommendations and precautionary measures are given below:

##### Recommendations for animal health professionals

- To maintain animal production and prevent possible adversities, it is necessary to ensure the sustainability of ordinary processes such as feed shipment and supply of feed raw materials by applying the necessary disinfection processes (Table 2). It is also important to respect physical distancing and hygiene practices recommended by the WHO (17) to avoid human-to-human transmission.
- The animal health professionals should be informed (from trusted sources) of the evolution of the COVID-19 pandemic in particular in their locality and of the clinical and therapeutic progress of the disease to implement new preventive methods and biosafety measures if necessary.
- Anyone with COVID-19 symptoms (confirmed or suspected cases) including asymptomatic or recovering patients should avoid close contact with animals until cleared by medical providers.
- Prevent animal diseases by maintaining good animal husbandry and production practices.
- Workers, especially veterinarians, should have a contingency plan, such as keeping an inventory of chemicals and equipment and implementing the latest laws and regulations regarding online veterinary consultation during the COVID-19 crisis.
- Animal health professionals should collaborate with pharmaceutical industries, communication channels (reliable social media, radio, and television), and staff in charge of the prevention and treatment of COVID-19 to organize meetings and seminars, preferably webinars, to raise awareness in animal production with important recommendations that can stabilize the activity.

**Table 2:** Disinfectants generally used against SARS-CoV-2 (COVID-19) to date (95, 96).

**Disinfectants**	**Solution**
Ethanol	At least 70%
Glutardialdehyde	0.5–2.5%
Povidone iodine	0.5%
Sodium hypochlorite	0.1% sodium hypochlorite, which can be made by 1:50 dilution of household bleach (5.25–6% sodium hypochlorite)
Hydrogen peroxide	3%

##### Recommendations to improve the economy

- The countries should maintain open borders for imports and exports to allow trade while respecting all COVID-19 preventives measures.
- The government should provide financial means or loans to animal production centers to increase the supply of animal feed and pay the wages of workers.
- The government should promote alternative sales channels of animal products such as e-commerce instead of public markets to avoid physical contact between people to prevent disease transmission and boost the economy.
- Develop an alternative and effective strategy to increase production with fewer workers, for example by adjusting working arrangements if an employee is absent due to COVID19 or in quarantine and by adjusting the sick leave policy of workers.
- To ensure the optimal functioning of the animal products supply chain, governments can promote collective marketing to maintain demand, and coordinate with suppliers to buy products and redistribute them through adequate channels (for example organizations such as UNICEF, UNHCR, etc.).

## Conclusion

The increasing prevalence of COVID-19 infection worldwide is a serious international public health issue as it affects all aspects of life. Although some countries are reopening, the number of confirmed COVID-19 cases is still increasing day by day and the second phase of contamination is observed in many countries. Some researchers have proposed the use of some vitamins (vitamin A, C, D, and E) and foods to boost the immune system and prevent COVID-19 infection, but a specific effective treatment or vaccine against COVID-19 is not yet available. It is imperative to follow the preventive methods recommended by the WHO to limit the spread of this disease. Recent data reported the possibility of transmission from infected patients to animals, so adequate surveillance is also needed to detect infected animals and workers at an early stage for efficient response. Although there is no evidence of disease transmission through the foodstuff or its packaging, it is important to reinforce the current hygiene rules and to make them more attentive to eliminate concerns in consumers' approach to the consumption of foodstuffs. To meet the demand for animal products and boost the economy, it is essential to resolve access to animal feed and other raw materials on a state basis.

## Author Contributions

PBDD, VK, CO, and KS participated in the study conception, implementation and data analysis, and drafted the manuscript. KS supervised this study from the conception to manuscript review and validation. All co-authors read and approved the final manuscript.

## Conflict of Interest

The authors declare that the research was conducted in the absence of any commercial or financial relationships that could be construed as a potential conflict of interest. The handling editor declared a past co-authorship with one of the authors KS.

## Footnotes

^1^https://clinicaltrials.gov/ct2/results?cond=covid-19&term=dexamethasone&cntry=&state=&city=&dist= (accessed September 23, 2020).

^2^https://www.mpg.de/14663263/artemisia-annua-corona-virus (accessed June 24, 2020).

^3^https://www.aphis.usda.gov/aphis/ourfocus/animalhealth/sa_one_health/sars-cov-2-animals-us (accessed September 23, 2020).

^4^https://www.cdc.gov/coronavirus/2019-ncov/animals/animal-testing.html (accessed September 23, 2020).

^5^http://yem.org.tr (accessed July 23, 2020).

^6^www.gov.uk/government/statistical-data-sets/animal-feed-prices (accessed July 23, 2020).

^7^https://www.pigprogress.net/World-of-Pigs1/Articles/2020/4/How-are-pig-producers-aroundthe-world-affected-by-Covid-19-568258E/ (accessed July 8, 2020).
